# Bootstrapping of Parameterized Skills Through Hybrid Optimization in Task and Policy Spaces

**DOI:** 10.3389/frobt.2018.00049

**Published:** 2018-06-08

**Authors:** Jeffrey F. Queißer, Jochen J. Steil

**Affiliations:** ^1^Research Institute for Cognition and Robotics (CoR-Lab), Machine Learning Group, CITEC, Bielefeld University, Bielefeld, Germany; ^2^Institute for Robotics and Process Control, Technische Universität Braunschweig, Braunschweig, Germany

**Keywords:** reinforcement learning, policy optimization, memory, learning, hybrid optimization, dimensionality reduction, parameterized skills

## Abstract

Modern robotic applications create high demands on adaptation of actions with respect to variance in a given task. Reinforcement learning is able to optimize for these changing conditions, but relearning from scratch is hardly feasible due to the high number of required rollouts. We propose a parameterized skill that generalizes to new actions for changing task parameters, which is encoded as a meta-learner that provides parameters for task-specific dynamic motion primitives. Our work shows that utilizing parameterized skills for initialization of the optimization process leads to a more effective incremental task learning. In addition, we introduce a hybrid optimization method that combines a fast coarse optimization on a manifold of policy parameters with a fine grained parameter search in the unrestricted space of actions. The proposed algorithm reduces the number of required rollouts for adaptation to new task conditions. Application in illustrative toy scenarios, for a 10-DOF planar arm, and a humanoid robot point reaching task validate the approach.

## 1. Introduction

Advanced robotic systems face non-static environmental conditions which require context-dependent adaptation of motor skills. Approaches that optimize motions for a given task by reinforcement learning, like object manipulation (Günter, 2009) or walking gait exploration (Cai and Jiang, 2013), deal only with a single instance of a potentially parameterized set of tasks. In many cases, a low-dimensional parameterization that covers the variance of a task exists. For example, consider reaching and grasping under various obstacle positions and object postures (Ude et al., 2007; Stulp et al., 2013), throwing of objects at parameterized target positions (Silva et al., 2014) or playing table tennis using motion primitives that are parameterized with respect to the current ball trajectory (Kober et al., 2012). A full optimization for each new task parameterization from a reasonable initialization, which was acquired by e.g., kinesthetic teaching, means that many computations and trials need to be performed before the task can be executed. This impedes immediate task execution and is highly inefficient for executing repetitive tasks under some structured variation.

Recent work addresses this issue by introducing parameterized motor skills that estimate a mapping between the parameterization of a task and corresponding solutions in policy parameter space (Ude et al., 2007; Mülling et al., 2010; Matsubara et al., 2011; Kober et al., 2012; Baranes et al., 2013; Stulp et al., 2013; Silva et al., 2014; Reinhart and Steil, 2015). Generation of training data for the update of such parameterized skills requires the collection of optimized policies for a number of task parameterizations. In previous work, each training sample is based on a full optimization for a new task parameterization starting from a fixed initialization (Silva et al., 2012, 2014), or gathered in demonstrations e.g., by kinesthetic teaching (Ude et al., 2007; Matsubara et al., 2011; Stulp et al., 2013; Reinhart and Steil, 2015). On the one hand, requesting demonstrations from a human teacher for many task parameterizations is not only time-consuming, but also includes the risk of collecting very different solutions to similar tasks due to the redundancy of the problem. Solutions on a smooth manifold are a prerequisite to allow for generalization for unknown tasks by machine learning algorithms. On the other hand, full optimization from a single initial condition requires many rollouts and ignores the already acquired knowledge about the motor skill.

In this work, we follow the idea of (Silva et al., 2012, 2014; Baranes et al., 2013) to apply dedicated policy optimization for new task parameterizations instead of gathering demonstrations from a tutor. In a similar way as (Baranes et al., 2013), we generalize for new task parameterizations to transfer optimization results. We investigate an incremental algorithm to establish parameterized skills that reuse previous experience to successively improve the initialization of the optimization process (Queißer et al., 2016). Thereby we are able to incorporate state-of-the-art optimization of the policy, i.e., by CMA-ES, instead of optimizing meta-parameters of policies (Kober et al., 2012) and do not rely on library based approaches (Mülling et al., 2010). In contrast to (Silva et al., 2012, 2014), the optimizer is initialized with the current estimate of the iteratively updated parameterized skill. The parameterized skill is a meta learner for generalization of DMP parameterizations for given task parameterizations. This leads to a significant reduction of the number of required rollouts during skill acquisition. We refer to the process of incremental skill acquisition as *bootstrapping*. We systematically show that the optimization process benefits from the initial condition proposed by the not yet fully trained parameterized skill and how this benefit depends on the model complexity of the learning algorithm. To cope with redundancy and to support the exploration of smooth manifolds in the policy parameter space, we introduce an additional cost term for optimization that we refer to as *regularization* of policy parameterization. In addition, we apply ridge regression with regularization for estimation of a smooth parameterized skill representation. The proposed algorithm for bootstrapping of parameterized skills results in a significant speed-up of the optimization processes for novel task parameterizations.

**Figure 1:**
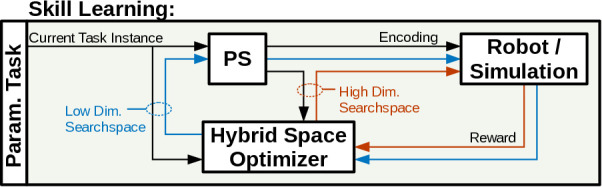
Hybrid optimization framework, the optimizer is initialized (H1) by the current estimate (gray) of the parameterized skill PS and performs a hybrid optimization (H2) in the low dimensional manifold of previous solutions (blue) and the high dimensional space of motion primitives (red).

Based on previous experiments (Queißer et al., 2016), we argue for a utilization of the parameterized skill as a projection of the low dimensional manifold of task-space to the high dimensional search space of policy parameters. By performing a policy optimization in this low dimensional manifold, a further speed-up, in terms of number of rollouts, during the optimization process can be observed. But to cope with the very likely case that no sufficient solution for the required task can be found in the manifold of the parameterized skill, we propose to perform a hybrid search in both spaces. Therefore we introduce an hybrid optimization algorithm that samples rollouts in both spaces and performs an estimation for a combined parameter update, as outlined in Figure 1.

This work extends our previous method (Queißer et al., 2016) and its contribution aims at the experimental verification of the following hypotheses:

(H1) Initialization of the optimizer with the current estimate of the parameterized skill leads to a faster optimization and convergence of the skill learning. (Sec. 3)

(H2) Searching in the manifold of previous solutions leads to a reduction of search space and thereby to a more efficient acquisition of the parameterized skill. (Sec. 4)

**Figure 2:**
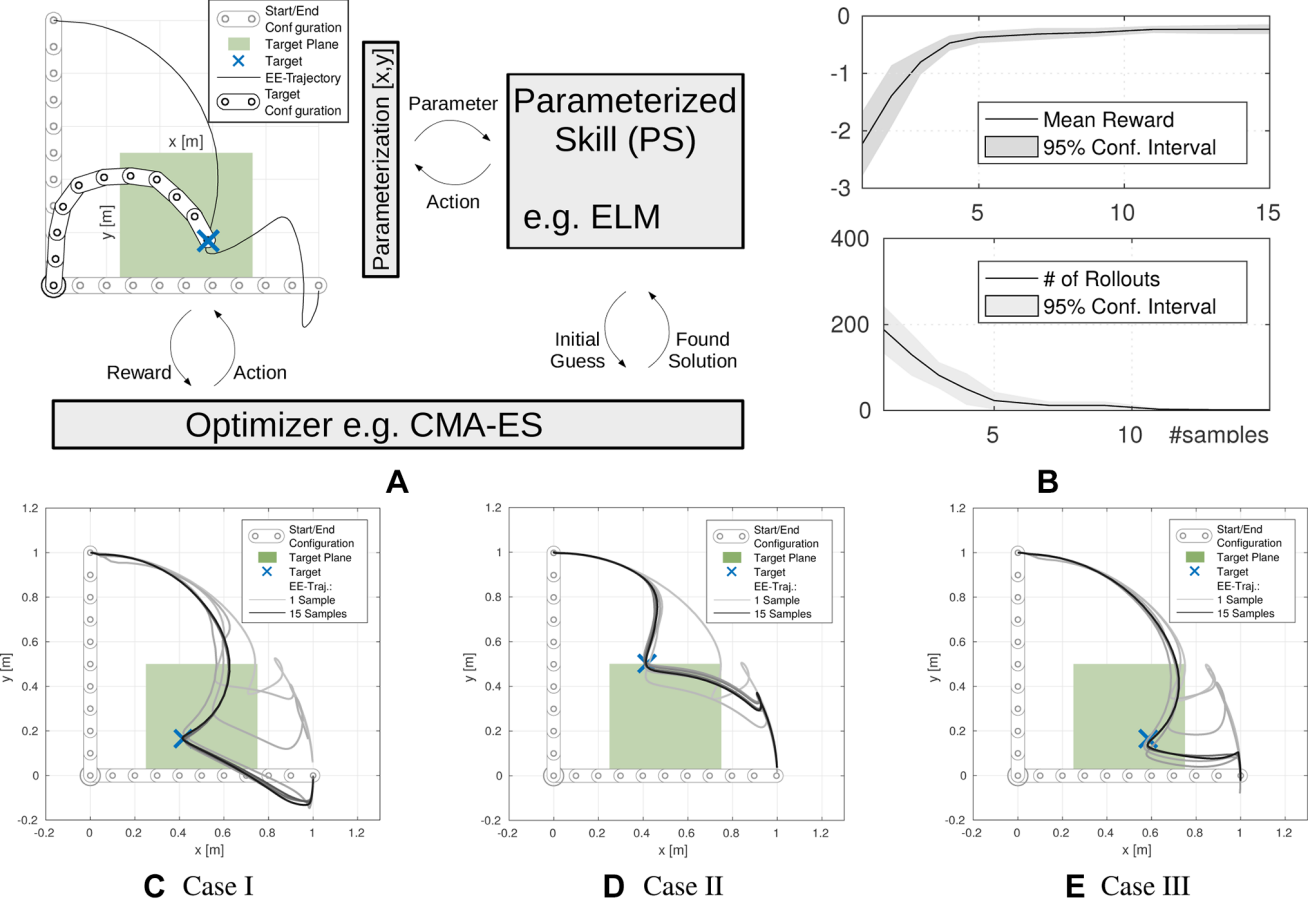
Bootstrapping loop of parameterized skills as proposed in (Queißer et al., 2016). **(A)** System overview including simulation of a 10-DOF planar arm, parameterized reaching target at $\frac{T}{2}$, parameterized skill and optimization module. **(B)** Result of the bootstrapping experiment, as more samples have been presented to the parameterized skill, the higher the initial reward and the lower the number of rollouts to fulfil unseen tasks. In **(C)**-**(E)**, three exemplary test cases for *τ* are shown. The representation of the parameterized skill in relation to the number (gray scale) of consolidated samples is visualized. © 2016 IEEE, reproduced with permission from (Queißer et al., 2016).

**Figure 3:**
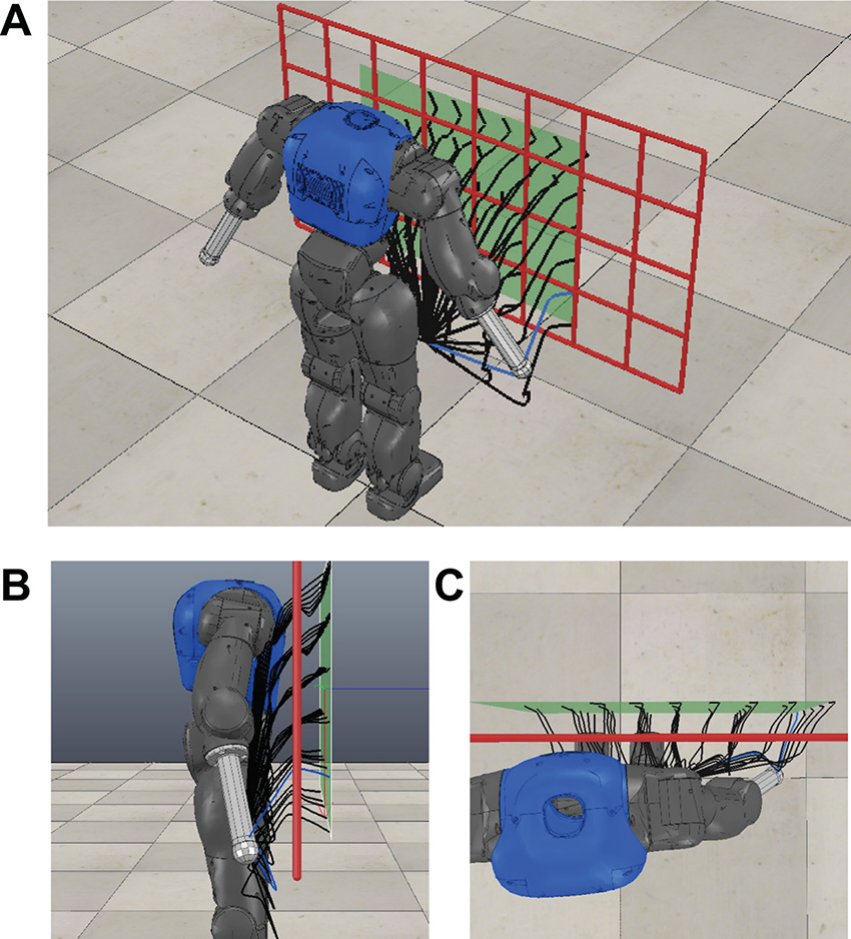
Constrained reaching scenario with a humanoid upper body and a grid-shaped obstacle. Generalized end-effector trajectories for different reaching targets retrieved from the iteratively trained parameterized skill are shown by black lines. © 2016 IEEE, reproduced with permission from (Queißer et al., 2016).

We evaluate the bootstrapping and the hybrid search of the proposed algorithm on a via point task with a planar 10-DOF robot arm (see Figure 2). Additionally we investigate the properties of the proposed optimization in hybrid spaces on toy examples. The scalability of the approach is demonstrated by bootstrapping a parameterized skill for a reaching task incorporating the upper body kinematics of the humanoid robot COMAN (see Figure 3) in end-effector as well as joint space control.

## 2. Parameterized Skills

**Figure 4:**
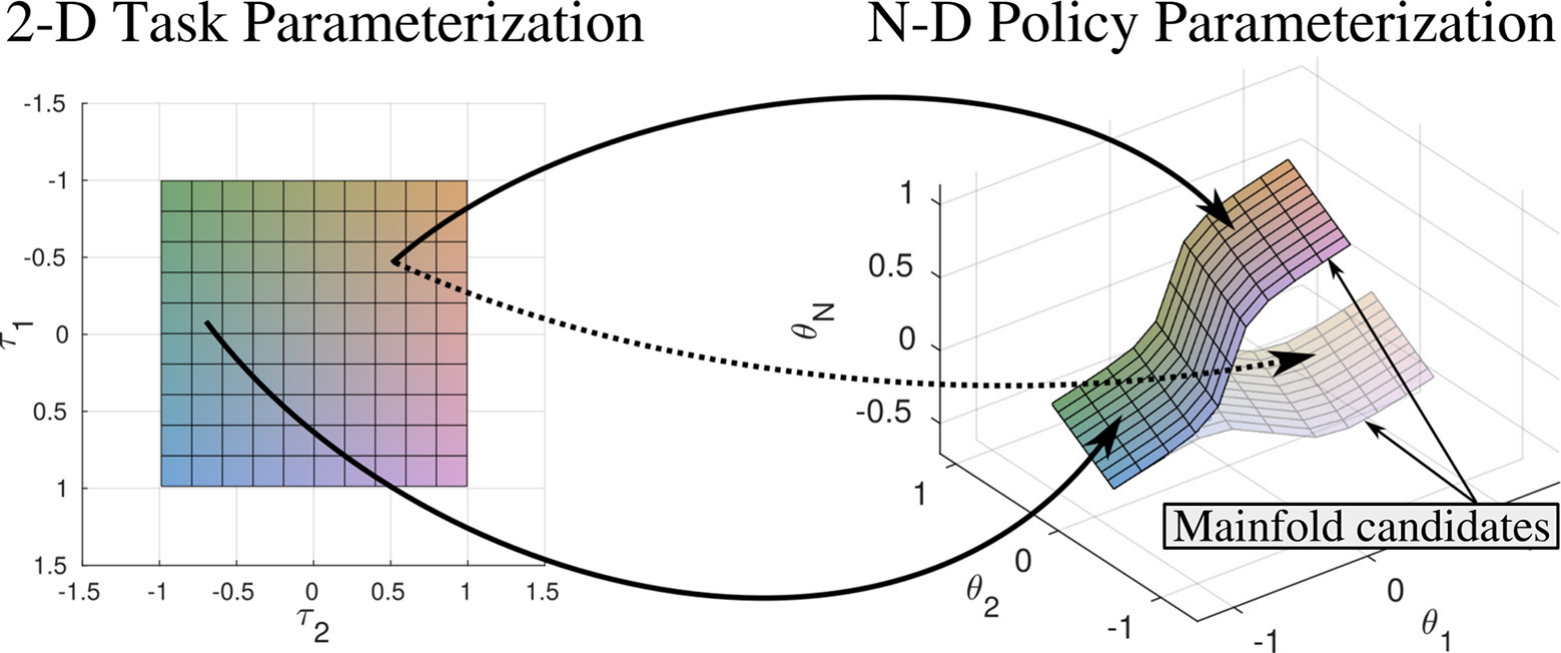
We expect that multiple manifolds exist that are suitable to describe a given task. Therefore the estimation of policy parameterizations that lie close to only one of the manifold candidates allows to estimate a smooth mapping between task and policy parameterization. Policy parameterizations that originate from different manifold candidates can result in ambiguous training data and decrease generalization capabilities of the parameterized skill. Coloring indicates mapping from input space to position on manifold.

We consider policies *π_**θ**_* that are parameterized by ***θ***∈ ℝ*^F^*. We further assume that tasks are parameterized by ***τ***∈ ℝ*^E^*. Task instances defined by ***τ*** are distributed according to the probability density function *P*(***τ***). The task parameterization ***τ*** reflects the variability of the task, e.g., position of obstacles, target positions or load attached to an end-effector. With reference to (Silva et al., 2014), we introduce the notion of a parameterized skill, which is given by a function PS : ℝ*^E^* → ℝ*^F^* that maps task parameters ***τ*** to policy parameters ***θ***. The goal is to find a parameterized skill PS(***τ***) that maximizes $\int P\left(\tau \right)J\left(\pi_{\text{PS}\left(\tau \right),}\tau \right)d\tau$ where *J*(*π*, ***τ***) = *E* {*R*(*π_**θ**_***, *****τ***)|*π*, ***τ***} is the expected reward for using policy *π_**θ**_* to solve a task ***τ***. The reward function *R*(*π_**θ**_*, *τ*) assesses each action of the robot defined by the policy *π_**θ**_* with respect to the current task parameterization ***τ***. In Figure 4 we visualize the relation between task space and the policy parameterization. We expect that multiple manifolds for a given task parameterization exist. We therefore have to select one of the candidated manifolds for approximation by the parameterized skill. We support the incremental exploration of a continuous mapping between ***τ*** and ***θ*** by imposing a respective preference for solutions that are close to the current estimate of the parameterized skill.

## 3. Bootstrapping of Parameterized Skills

We propose an algorithm to bootstrap a parameterized skill PS(***τ***) by consolidating optimized ***θ*** for given ***τ***. We assume that some sort of policy representation, e.g., a motion primitive model, and policy search algorithm, e.g., REINFORCE (Williams, 1992) or CMA-ES (Hansen, 2006), are available. The idea is to incrementally train the parameterized skill PS(***τ***) with task-policy parameter pairs (***τ***, θ*), where θ* are optimized policy parameters obtained by executing the policy search algorithm for task ***τ***. The key step is that the current estimate PS(***τ***) of policy parameters is used as initial condition for policy optimization of new tasks ***τ***. With reference to hypothesis H1 of Sec. 1, the central outcome of this procedure is that policy search becomes very efficient due to incrementally better initial conditions of the policy search. Ultimately, PS(***τ***) directly provides optimal policy parameters and no further policy optimization needs to be conducted.

**Figure 5:**
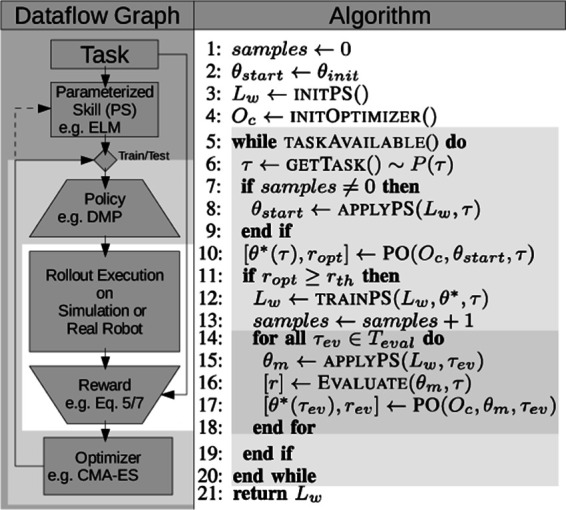
Dataflow and pseudocode of the proposed bootstrapping algorithm. The parameterized skill (PS) estimates a policy parameterization ***θ**_start_*. In case of training, successive policy optimization (PO) by reinforcement learning results in an update of the parameterized skill. The shading of the background highlights nested processing loops of the system (from outer to inner): (1) Iteration over all tasks; (2) Optimization of ***θ*** by the PO algorithm; (3) Execution and estimation of the reward by iterating over all *T* timesteps of the trajectory $p_{t}^{*}$. © 2016 IEEE, reproduced with permission from (Queißer et al., 2016).

The algorithm for the parameterized skill acquisition is outlined in Figure 5. For each new task *τ*, the parameterized skill provides an initial policy parameterization ***θ**_start_* =  startPS(***τ***) (line 8). After collecting a sufficient number of pairs (***τ***, θ*), the proposed parameterization ***θ**_start_* can achieve satisfactory rewards such that no further policy optimization (PO) by reinforcement learning is necessary. The optimization from initial condition PS(***τ***) is initiated if the estimated policy parameters can not yet solve the given task or further training is desired (line 10). To ensure that only successful optimization results are used for training of the parameterized skill, an evaluation of the optimization process (e.g., reward *r_opt_* exceeds a threshold *r_th_*) is performed (line 11). If the optimization was successful, the pair (***τ***, θ*) with optimized policy parameters θ* is used for supervised learning of PS(***τ***) (line 12). Finally, lines 14–18 serve evaluation purposes during incremental training. The evaluation was performed on a predefined set of evaluation tasks in ***τ**_ev_*∈ *T_ev_* that are disjunct from the training samples.

### 3.1. Selection of Policy Representation

The proposed method does not rely on a specific type of policy representation. Many methods for compact policy presentation have been proposed, e.g., based on Gaussian Mixture Models (GMM) (Günter et al., 2007) or Neural Imprinted Vector Fields (Lemme et al., 2014). We employ Dynamic Motion Primitives [DMP, (Schaal, 2006; Ijspeert et al., 2013)], because they are widely used in the field of motion generation. DMPs for point-to-point motions are based on a dynamical point attractor system

(1) $$\ddot{y}=k_{S}\left(g-y\right)-k_{D}\ddot{y}+f\left(x,\theta \right)$$

that defines the output trajectory as well as velocity and acceleration profiles. The canonical system is typically defined as

$\dot{x}=-\alpha x$ or in our case as a linear decay $\dot{x}=-\alpha$ as in (Kulvicius et al., 2012). The shape of the primitive is defined by

(2) $$f\left(x,\theta \right)=\frac{\sum_{k=1}^{K}exp\left(-V_{k}\left(x-C_{k}\right)\right)\theta_{k}}{\sum_{k=1}^{K}exp\left(-V_{k}\left(x-C_{k}\right)\right)},$$

where a mixture of *K* Gaussians is used. ***C**_k_* are the Gaussian centers and ***V**_k_* define the variance of the Gaussians. The DMP is parameterized by the mixing coefficients *θ_k_*. We assume fixed variances ***V**_k_* and a regular spacing of centers ***C**_k_* as in (Ijspeert et al., 2013; Reinhart and Steil, 2015).

### 3.2. Selection of Policy Optimization Algorithm

We apply the Covariance Matrix Adaptation Evolutionary Strategy [CMA-ES, (Hansen, 2006)] for optimization of DMP parameters ***θ***, given a task ***τ***. Stulp et al. (Stulp and Sigaud, 2013) have shown that the black-box optimization by CMA-ES is very efficient and reliable in combination with DMPs. In comparison to other reinforcement learning methods like PI^2^ (Theodorou et al., 2010) or REINFORCE (Williams, 1992), which evaluate the reward at each time step, CMA-ES is a black-box-optimization algorithm and operates only on the total reward of an action sequence. Stochastic optimization by CMA-ES evaluates *N_λ_* rollouts of policy parameters per generation, which are drawn from a Gaussian distribution centered at the current policy parameter estimate. For each generation the current estimate is updated by a weighted mean of all *N_λ_* rollouts. The final number of rollouts *R* required for optimization is given by the number of generations times the number *N_λ_* of rollouts per generation.

### 3.3. Selection of Learning Algorithm

For learning of parameterized skills PS(***τ***), we apply an incremental variant of the Extreme Learning Machine [ELM, (Huang et al., 2006)]. ELMs are feedforward neural networks with a single hidden layer:

(3) $$\theta_{i}\left(\tau \right)=\sum_{j=1}^{H}{\text{W}}_{ij}^{out}\sigma \left(\sum_{k=1}^{E}{\text{W}}_{jk}^{inp}\tau_{k}+b_{j}\right)\;\forall i=1,\;\ldots ,N$$

with input dimensionality *E*, hidden layer size *H* and output dimensionality *F*. Hidden Layer size was set to *H* = 50 for generalization in joint space and *H* = 20 in case of Cartesian end-effector space. Regression is based on a random projection of the input **W***^inp^* ∈ ℝ*^H×E^*, a non-linear transformation *σ*(*x*) = (1 + *e^–x^*)^–1^ and a linear output transformation **W***^out^*∈ ℝ*^F×H^* that can be updated by incremental least squares algorithms. The incremental update scheme of the ELM was introduced as Online Sequential Extreme Learning Machine (OSELM) (Liang et al., 2006) that incorporates the ability to perform an additional regularization on the weights (Huynh and Won, 2009) or exponential forgetting of previous samples (Zhao et al., 2012). Since we expect to deal with a small number of training samples, regularization of the network can help to prevent over-fitting and foster reasonable extrapolation.

### 3.4. Experiments

The experimental setup for evaluation of our proposed bootstrapping architecture is shown in Figure 2A. The optimization algorithm is initialized by an *initial guess* of the parameterized skill. During optimization the optimizer performs rollouts in simulation and observes rewards resulting of the requested actions. In case an appropriate action that fulfils the task could be found, an update of the parameterized skill is conducted as explained previously in detail in Sec. 3

#### 3.4.1. 10-DOF Planar Arm via-Point Task

The goal is to optimize the parameters of a DMP policy to generate joint angle trajectories such that the end-effector of a 10-DOF planar arm passes through a via-point in task space at time step $\frac{T}{2}$ of the movement with duration *T*. We considered the kinematics of a 10-DOF planar arm. Motions start at initial configuration ***q**_start_* = (0, 0, 0, 0, 0, 0, 0, 0, 0, 0)^T^ and stop at configuration ***q**_end_* = (π/2, 0, 0, 0, 0, 0, 0, 0, 0, 0)^T^. The task parameterization ***τ*** is given by the 2D via-point position ***τ***= (***v**_x_, **v**_y_*) of the end-effector at timestep $\frac{T}{2}$.

. The task parameterization ***τ*** is given by the 2D via-point position ***τ***= (***v**_x_, **v**_y_*) of the end-effector at timestep $\frac{T}{2}$.

Since there exists no unique mapping between task and policy parameter space in this example, infinite action parameterizations can be found that sufficiently solve a given task (e.g., exceed a reward threshold). To reduce ambiguities in the training data for parameterized skill learning, we add a *policy regularization* term to the reward function. This *regularization* punishes the deviation of the policy parameters PS(***τ***) from the initial parameters ***θ**_init_* and additionally rewards small jerk of the end-effector trajectory. The initial and final arm configurations are shown in Figure 2. We utilize a minimum jerk trajectory (Flash and Hogan, 1985) in joint angle space to generate the initial policy parameters ***θ**_init_*.

The overall reward *R*(***θ**, **v***) is given by:

(4) $$\begin{array}{rl} R\left(\theta ,v\right)=-\underset{\text{Jerk (a)}}{\underbrace{\alpha_{1}\sum_{t=2}^{T}{\left(\frac{\partial^{3}p_{t}^{x}}{\partial t^{3}}\right)}^{2}+{\left(\frac{\partial^{3}p_{t}^{y}}{\partial t^{3}}\right)}^{2}}} & -\underset{\text{Via Point (b)}}{\underbrace{\alpha_{2}\|p_{T/2}-v_{p}{\|}^{2}}}\\ & -\underset{\text{Regularization (c)}}{\underbrace{\alpha_{3}\|\theta_{\text{init}}-\theta \|}} \end{array}$$

The reward depends on the DMP parameters ***θ*** that result in a 10 dimensional joint trajectory transformed by the kinematics of the robot arm to the end-effector trajectory ***p**_t_*. The jerk is based on the third derivative of the end-effector trajectory ***p**_t_* (Hogan, 1984; Flash and Hogan, 1985) and is represented as one objective in the reward function Equation 4(a). Additional terms of the reward function refer to the distance to the desired via-point *v* of the end-effector trajectory Equation 4(b) and the regularization Equation 4(c).

Coefficients *α_i_* are fixed for all experiments to ***α*** = (10^2^, 15, 10^–3^)^T^, resulting in a magnitude of the regularization of ca. 10% of the overall reward of an optimized task. For the training phase, we selected *N_train_* = 15 random tasks ***τ***, i.e., via-point positions drawn from the green target plane in Figure 2. Generalization performance is evaluated on a fixed test set ***τ**_ev_* of *N_test_* = 16 via-points arranged in a grid on the target plane. For each of the 10 joints of the robot, we selected a DMP with *K* = 6 basis functions, resulting in a *F* = 60 dimensional policy parameterization ***θ***. On the top of Figure 2B, the mean initial reward for all tasks ***τ**_ev_* in the test set is shown. The initial reward is based on the reward for estimated policy parameters PS(***τ***) as function of the number of incorporated training samples. The lower part of Figure 2B shows that policy optimization benefits from the improved initial policy parameters PS(***τ***) by reducing the number of required rollouts to solve novel tasks (exceed a certain reward threshold). The results show a significant reduction of the required number of rollouts compared to the initialization with the first training sample θ*, that is regarded as the baseline. Figure 2C-E shows solutions for three exemplary tasks *τ* from the test set. The gray scale of the end-effector trajectories refers to the number of consolidated training samples and shows that the parameterized skill improves as more optimized samples have been used for training. Further evaluation of the properties of the parameterized skill can be found in preliminary work (Queißer et al., 2016), this includes a comparison of different learner and exponential forgetting of old training data.

#### 3.4.2. Reaching Through a Grid

The scenario shows the scalability of the proposed approach to more complex tasks. The goal is to reach for variable positions behind a grid-shaped obstacle while avoiding collisions of the arm with the grid as well as self-collisions. The experiments are performed in simulation of the humanoid robot COMAN (Tsagarakis et al., 2011) as shown in Figure 3. We control 7 DOF of the upper body including waist, chest and right arm joints. For the first part of the experiment, motions are represented in Cartesian space utilizing 3 DMPs with *K* = 5 basis functions (as in Equation 2), resulting in a *F* = 15 dimensional optimization problem. The respective DMPs are executed yielding Cartesian end-effector trajectories $p_{t}^{*}$. As before, we utilize minimum jerk trajectories to generate the initial policy parameters ***θ**_init_*. The subset of valid and executable end-effector trajectories ***p**_r,t_* in Cartesian space is given by the kinematics as well as the reachability (e.g., joint limits) of the robot joints.

For each time step *t* of the desired end-effector trajectory $p_{t}^{*}$, an inverse Jacobian controller estimates a configuration of the robot that executes $p_{t}^{*}$ and maximizes the distance to all obstacles in the null-space of the manipulator Jacobian (Liegeois, 1977):

(5) $$\dot{q}=J^{†}\left(p_{t}^{*}-p_{r,t}\right)+\alpha \left(I-J^{†}J\right)Z$$

(6) $$Z=\sum_{l=1}^{L}-J_{p,l}^{\text{T}}\cdot d_{min,l}$$

where $p_{t}^{*}-p_{r,t}$ is the distance between the desired end-effector trajectory $p_{t}^{*}$ and the trajectory ***p**_r,t_* reached by the robot. The term *Z* maximizes the distances $\left\|d_{min,l}\right\|$ of all *L* links to the grid obstacle in the null-space I – ***J***^†^***J***. The maximization by following the direction –***d**_**min,l**_* in joint space is implemented by the point Jacobian $J_{p,l}^{\text{T}}$ of the closest point to the obstacle. The reward function for policy optimization is given by:

(7) R(θ,vp)=−α1∑t=2T‖pt∗−pt−1∗‖⏟Length of Trajecory (a)−α2∑t=1T‖pt∗−pr,t‖⏟Task Tracking (b)+α3∑t=1Trd,t⏟Dist. to Obstacles (c)−∑tTα4‖θinit−θ‖⏟Regularization (d)

where *T* is the duration of the trajectory. The reward in Equation 7 is a weighted sum of four objectives with weighting factors *α_i_*: (a) The length of the desired end-effector trajectory ***p**_d,t_* that is defined by policy parameter *θ* [Equation 7(a)]; (b) In addition to the punishment of long trajectories, the reward takes the accuracy of the trajectory tracking into account. Therefore, Equation 7(b) punishes deviations of the reached end-effector position ***p**_r,t_* in relation to the desired end-effector position $p_{t}^{*}$; (c) The distance maximization of all links to the grid obstacle *r_d,t_* is considered in Equation 7(c). The optimization criterion representing the maximization of the distance to the grid-obstacle *r_d,t_* is given by:

(8) $$r_{d,t}=-\sum_{l=1}^{L}min{\left(0,\;\|d_{min,l}\|-d_{B}\right)}^{2}$$

a quadratic relationship to the minimum distances ***d**_min,l_* over all *L* links to all obstacles in the scene in case the distance falls below a given threshold ***d**_B_*, as in (Toussaint et al., 2007) introduced in the context of null-space constraints for humanoid robot movement generation; (d) An additional normalization for small policy parameterizations as given by Equation 7(d).

The second part of the experiment refers to DMPs in joint space to represent the complete motion of the robot. Therefore the policy parameterization has to represent the maximization of the distance to the grid shaped obstacle as well since no additional inverse Jacobian controller is used. For this experiment we utilize 7 DMPs with *K* = 15 basis functions (as in Equation 2) that generate joint space trajectories, resulting in a 105 dimensional optimization problem. For policy optimization, the reward function is similar to that for the for end-effector trajectories Equation 7. The policy parameters are transformed by DMPs to desired joint space trajectories $p_{t}^{*}$. As previously introduced, Equation 7(b) reflects physical constraints of the robot like joint limits. We initialize ***θ***_init_ with joint angle trajectories that allow the end-effector to follow a straight line from start to goal position.

### 3.5. Results

**Figure 6:**
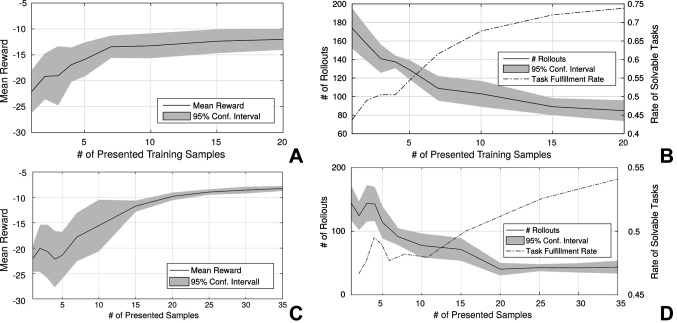
Mean reward of the initial guess ***θ**_start_* = PS(***τ***) of the parameterized skill in relation to the number of presented training samples **(A/C)** and the mean number of rollouts that are necessary to solve selected test tasks (reward exceeds a threshold) **(B/D)**. Figures **(A/B)** show results of the experiments in end-effector space whereas **(C/D)** show results of the experiments in joint space. The dashed line in **(B/D)** shows the mean rate of solvable task in the test set. Results and confidence intervals are based on ten repeated experiments. Figure **(A/B)** © 2016 IEEE, reproduced with permission from (Queißer et al., 2016).

We evaluated the bootstrapping of the parameterized skill as outlined in Figure 5. We selected *N_train_* = 20 random target positions on the target plane in front of the robot for training. For evaluation, we created a fixed regular grid for point sampling of *N_test_* = 39 positions on the target plane. Figure 6 reveals that the reward of the initial guess ***θ**_start_* = PS(*τ*) of the parameterized skill increases with the number of presented training samples. In comparison to the previous experiment in Sec. 3.4.1, the optimization algorithm does not always succeed to find a solution for all tasks of the test set. Figure 6B shows an increasing success rate in relation to the number of consolidated samples and thereby the reward of the initial parameters ***θ**_start_* of the policy search. This indicates that increasingly better initial conditions PS(***τ***) for policy optimization reduce the risk to get stuck in local minima during optimization. In terms of number of rollouts that are required to fulfil a new task, we observe similar results as in the 10-DOF arm experiment: The number of required rollouts necessary for task fulfilment decreases the more successfully solved task instances have been presented to the parameterized skill as training data. This results in a bootstrapping and acceleration of the parameterized skill learning. Although the experiments in end-effector space utilize a joint controller that maximizes the distances automatically, the system is able to achieve similar performance in joint space except of a slightly lower success rate.

## 4. Optimization in Hybrid Spaces

Sec. 3 showed that the utilization of a parameterized skill for policy optimization by CMA-ES can significantly reduce the number of rollouts required to solve unseen tasks. A further option to speed up policy search is given by policy optimization in a lower dimensional search space, as stated by hypothesis H2 of Sec. 1. Previous work of (Koutnik et al., 2010; Fabisch et al., 2013) has already demonstrated that a compression of the parameter space by use of multi layer perceptrons (MLPs) leads to an acceleration of optimization for reinforcement tasks. Reduction of the search spaces by manifolds for value function approximation (Glaubius and Smart, 2005) and abstraction of the whole state-space into sub areas for terrain navigation (Glaubius et al., 2005) can be beneficial in case of reinforcement learning. Constrained optimization problems have been tackled by reducing state-space evaluations and focus on the feasible space of parameters (Barkat et al., 2008). It was demonstrated that the reduction of the number of available bio-mechanical DOF helps stabilize the interplay between environmental and neural dynamics (Lungarella and Berthouze, 2002) for robotic tasks. Dimensionality reduction by freezing or synchronization of joints allows for faster skill acquisition, as shown by (Kawai et al., 2012). Further related work has elaborated the intrinsic dimensionality of human movements and demonstrated that dimension reduction is beneficial for reinforcement learning on humanoid robot platforms (Colome et al., 2014).

We assume that previously optimized solutions (***τ***, θ*) represent the variability in the task domain and are consolidated in the parameterized skill. We therefore propose to reconsider the parameterized skill as an embedding *f***_emb_** of a non-linear manifold of task relevant actions within the full policy space *f*_PS_ : ℝ*^E^* → ℝ*^F^*, ***θ***_emb_ ↦ PS(***θ***_emb_). Further, we expect that solutions for unseen tasks are located close to the manifold of the parameterized skill, since we can observe the relation between a higher number of consolidated samples and a higher initial reward, as shown in Figures 2, 6. It is thus reasonable to perform policy optimization in the input space of *f*_PS_, due to the lower dimensionality of our task parameterization compared to the policy parameterization.

We expect that for points on *f*_PS_ and their local neighborhood a differentiable map, i.e., a chart of the manifold in the policy space, exists. But on a global scale, it can be expected that the mapping between the task space and the policy space is not invertible since different task parameterizations ***τ*** may require the same policy parameterization ***θ*** and is not differentiable due to e.g., joint limits. Previous work related to our proposed method for dimensionality reduction for policy optimization includes primitive based motion generation by PCA compression (Park and Jo, 2004), lower dimensional primitives that encode differences between trajectories (Stulp et al., 2009) and further library based approaches like (Moro et al., 2012).

But clearly, a search in the task space depends heavily on the number and quality of previously seen samples. We can not expect to be able to find sufficient solutions for all unseen tasks configurations on a low dimensional manifold. More specifically, an exploration on the approximated manifold allows for a coarse search that quickly moves the estimation for θ* into the direction of higher rewards. If we are not able to fulfill the given task or we are less efficient to find a better solutions in the task space, we are forced to switch to a slower refinement search in the policy space. But also a temporary switch back from a search in the policy space to the task space would be possible.

Since optimization in policy space is not bound to the manifold of *f*_PS_, the joint update between of both spaces requires an inverse estimate of the parameterized skill. We define the local inverse of PS as:

(9) $${\hat{\text{PS}}}^{-1}\left(\theta \right)=\underset{\tau }{min}\|PS\left(\tau \right)-\theta \|,$$

which allows to estimate a point on *f*_PS_ that gives the closest response for a desired output *θ*.

Our approach allows to combine rollouts performed in both spaces for an update of the optimization algorithm. We propose to refer to the success rate of the policies sampled in the respective spaces as defined in Sec. 4.1 for the estimation of the importance of each space during optimization. In general, our combination of optimizers is not bound for a specific optimization algorithm, for this work we refer to a hybrid CMA-ES approach as introduced in Sec. 4.1.

From a policy optimization considering both spaces, we expect the following advantages: First, we expect the algorithm to utilize a low dimensional manifold based on previous samples to perform a fast optimization followed by an optional successive full optimization. Second, by exploration of the manifold based on the parameterized skill, we assume to find solutions that fit to the current estimate of *f*_PS_. Therefore, we expect to enhance the consistency of the training data of the parameterized skill for complex reward functions that allow for multiple solutions in policy space. Sections Sec. 5-6 will validate these assumptions. We will visualize and discuss on the ideas on toy data sets and perform algorithm comparisons to CMA-ES in one space.

### 4.1. CMA-ES in Hybrid Spaces

We refer to CMA-ES (Hansen, 2006) for the implementation of the proposed hybrid optimization method. The original algorithm of CMA-ES relies on four main steps, detailed information can be found in Appendix A. Optimization is performed in generations, which means that an action has to be performed under several perturbations and based on the observation of rewards an updated mean is estimated. CMA-ES has an internal representation of the current mean and of the covariance matrix that allows for sampling of new actions normally distributed around the current mean. In addition, CMA-ES estimates an evolution path for the mean and the covariance matrix update. Those evolution paths allow for more stability to outliers and noise. The first step performs the sampling from a multivariate normal distribution centered at the current estimate (Equation A.1). Followed by the update of the estimated solution for the next generation with respect to the rewards of the sampled rollouts (Equation A.2). The third step targets the update of the covariance matrix and its evolution path (Equation A.3) and (Equation A.4). And the final step performs an update of the exploration width and its assigned evolution path (Equation A.5, Equation A.6).

**Figure 7:**
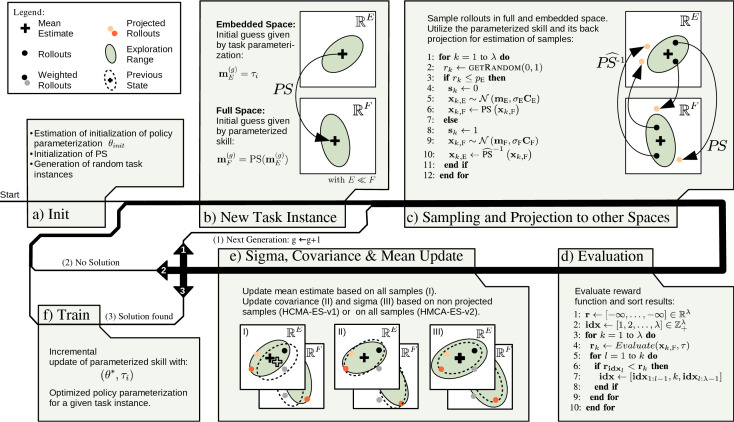
Proposed optimization loop for bootstrapping of parameterized skills in hybrid spaces. After initialization **(A)**, optimization for a new task instance is initiated **(B)**. Optimization is performed (1) until stopping criterion is reached and no solution was found (2) or the optimized solution fulfils the task (3). Update of CMA-ES (I-III) is performed for the task and policy space simultaneously.

To be able to perform CMA-ES in hybrid spaces, we apply the CMA-ES algorithm in two parameter spaces simultaneously. We add indices *F* and *E* to indicate the affiliation of variables for optimization in policy space (*F*) and task space (*E*). Two distinct means ${\text{m}}_{\text{E}}^{\left(g+1\right)}$ and ${\text{m}}_{\text{F}}^{\left(g+1\right)}$ represent the current optimum to minimize the objective function, i.e., negative reward. Covariance matrices ${\text{C}}_{\text{E}}^{\left(g+1\right)}$ and ${\text{C}}_{\text{F}}^{\left(g+1\right)}$ as well as their evolution paths ${\text{p}}_{c,\text{E}}^{\left(g+1\right)}$ and ${\text{p}}_{c,\text{F}}^{\left(g+1\right)}$ allow for random normal distributed perturbation of the respective mean. The variances $\sigma_{\text{E}}^{\left(g+1\right)}$ and $\sigma_{\text{F}}^{\left(g+1\right)}$ in addition to their evolution paths ${\text{p}}_{\sigma ,\text{E}}^{\left(g+1\right)}$ and ${\text{p}}_{\sigma ,\text{F}}^{\left(g+1\right)}$ define the exploration size in each space. In comparison to two independent CMA-ES optimizations in each space, we introduce a probability *p*_E_ respectively *p*_F_ = 1 – *p*_E_, that indicates in which space we sample the parameterization for rollouts. *p*_E_ and *p*_F_ can be interpreted as mixing coefficients that allow for interpolation between a CMA-ES optimization in the task space (*p*_E_ = 1) and a CMA-ES optimization in policy space (*p*_E_ = 0). For each update step we estimate $k=1,\;\ldots ,\;\lambda_{\text{H}}^{\left(g+1\right)}$ samples in generation (*g* + 1) for a combined update and annotation ${\text{s}}_{k}^{\left(g+1\right)}=0$ if rollout *k* was sampled in the task space or ${\text{s}}_{k}^{\left(g+1\right)}=1$ if sampled in the policy space. The initialization (Figure 7B) for a new task instance *i* of the parameterization in the embedded space is ${\text{m}}_{\text{E}}^{\left(g=0\right)}=\theta_{i}$ and the initialization in full space is given by the generalization of the parameterized skill ${\text{m}}_{\text{F}}^{\left(g+1\right)}=PS\left({\text{m}}_{\text{E}}^{\left(g=0\right)}\right).$ The sampling of rollouts as shown in Figure 7C is defined in (Equation B.1), for each rollout the target space for sampling is based on probabilities *p*_E_ and *p*_F_. The number of evaluated rollouts per generation is defined as a linear interpolation between *λ*_E_ and *λ*_F_ of both spaces as shown in Equation B.2. In a next step, the parameterized skill performs a mapping of samples originated in task space to policy space and vise versa (see Figure 7C, line 6 and 10). Therefore, a parameterized skill is required that allows for inverse evaluation notated as ${\hat{\text{PS}}}^{-1}$. This mapping process is given by Equation B.3. A representation of all rollouts in ${\text{x}}_{k,\text{F}}^{\left(g+1\right)}$ allows for execution of the policy and evaluation of the reward function. The rollouts are ordered based on the magnitude of the respective reward as proposed by the original CMA-ES approach (Hansen, 2006), as shown in Figure 7D. At this point an update of the means ${\text{m}}_{\text{E}}^{\left(g+1\right)}$ and ${\text{m}}_{\text{F}}^{\left(g+1\right)}$ with respect to ${\text{x}}_{k,\text{E}}^{\left(g+1\right)}$ and ${\text{x}}_{k,\text{F}}^{\left(g+1\right)}$ by applying Equation A.2 is possible. This allows for an update of the estimated means in both spaces based on all rollouts that have been evaluated in the current generation. Note, that the means ${\text{x}}_{k}^{\left(g+1\right)}$ do not develop independently. Rather they are linked by the mapping of the parametrized skill. For an adaptation of the ratio of rollouts performed in the policy and task space (*p*_F_ and *p*_E_), we utilize the success rate of both spaces. The success rate is defined as the ratio of successful rollouts (rollouts that exceed the current reward maximum), encoded by the weights ${\text{w}}_{k}^{\left(g+1\right)}$ as well as space that was used for sampling ${\text{s}}_{k}^{\left(g+1\right)}$ of the performed rollouts, as shown in Equation B.4.

We evaluate two approaches for an update of the covariance and exploration width: The first version utilizes only samples that originate in the same space for an update of the covariance C and exploration width *σ*. The second version utilizes the mapping of PS and ${\hat{\text{PS}}}^{-1}$ to estimate an additional update of the covariance and the exploration width with respect to all samples. We refer to the first version as **H**ybrid **C**ovariance **M**atrix **A**daptation - **E**volutionary **S**trategy - **V**ersion **1** (*HCMA-ES-v1*) and to the second version as *HCMA-ES-v2* .

#### 4.1.1. HCMA-ES-V1

The update of the covariance, exploration radius and their evolution paths is performed as in the original CMA-ES algorithm, depicted in Equation A.3–A.6. The update step for each space, encoded in ${\text{s}}_{k}^{\left(g+1\right)}$, considers only rollouts sampled in the same space. The normalization of $\sum {\text{w}}_{k}^{\left(g+1\right)}=1$ for all ${\text{s}}_{k}^{\left(g+a\right)}=0$ in case of the task space as well as ${\text{s}}_{k}^{\left(g+a\right)}=1$ in case of the policy space is necessary since not all samples are used in each space. Additionally, the estimation of *μ_e f f_*_,E_ and *μ_e f f_*_,F_ with respect to s has to be performed as well. The neglection of projected samples for the update of the covariance and its exploration width allows for simplification of the combination. But this simplification prevents that e.g., the covariance in the high dimensional policy space is able to shape into the direction of samples along the low dimensional manifold of the task parameterization.

#### 4.1.2. HCMA-ES-V2

The parameterized skill can be regarded as a mapping between the high dimensional policy parameterization and a low dimensional embedding. The shape of the multivariate normal distribution that is used for samples that get mapped to other spaces does not include a normal distribution in the target space due to the nonlinear transformation of the parameterized skill. For the integration of projected samples in the update of the covariance, we perform a rescaling of projected samples to cancel out the effect of the exploration width *σ*. The update of the exploration width *σ* requires the estimation of the distribution of projected samples, but the estimation of a covariance requires a much larger number of samples which is not feasible for our scenarios (≈ 10 rollouts). We decided for an update of exploration width $\sigma_{\text{E}}^{\left(g+1\right)}$ and $\sigma_{\text{F}}^{\left(g+1\right)}$ with respect to each other by the estimation of a scaling factor between the evaluated rollouts of the current generation which is applicable for low sample numbers.

To consider samples from other spaces for an update of the covariance and its evolution path, samples from other spaces have to be rescaled to keep the covariance *C* at a constant size i.e., *det* (**C***^T^***C**) = ∏*λ_i_ = const.*, the product of eigenvalues of **C** is constant. From the update of the exploration width given in Equation A.6 and Equation A.5, we are able to infer the condition $\sqrt{\mu_{e\;f\;f}}{\text{C}}^{{\left(g\right)}^{-\frac{1}{2}}}y_{w}=\text{E}\|N\left(0,\text{I}\right)\|$ for constant covariance size. Therefore, we add an appropriate scaling factor to the calculation of the weighted sum, resulting in a modified estimation of **ỹ**_w,E_ with an additional scaling of samples that originate in the full space Equation B.5. The estimation of **ỹ**_w,F_ is performed likewise, given by Equation B.6. The update of **p***_c_* and **C** can be achieved by Equation A.3 and Equation A.4 with respect to **ỹ**_w,E_ and **ỹ**_w,F_. The final step updates the exploration width $\sigma_{\text{E}}^{\left(g+1\right)}$ and $\sigma_{\text{F}}^{\left(g+1\right)}$. We achieve this by performing a mixing of the updated sigma of the own space and the rescaled sigma of the other space based on the success rate of the spaces (Equation B.7).

We will evaluate the properties of both algorithm versions and compare the results in successive experiments sections Sec. 5 and Sec. 6.

### 4.2. Implementation of the Parameterized Skill

The proposed optimization method does not rely on a specific learning method. But in comparison to the bootstrapping of the parameterized skills as proposed in Sec. 3, the policy search in hybrid spaces requires an inverse estimate of the parameterized skill. Therefore the learner must be continuous and locally differentiable. Candidates for this task are associative memories due to their intrinsic capabilities to be able to estimate an input for a given output of the mapping. For the evaluations presented in this paper we refer to a different approach, we utilize the Jacobian of the parameterized skill as proposed in Sec. 3 to iteratively estimate a proper input ***τ*** for a required output ***θ***. We refer to the Inverse Function Theorem by (Spivak, 1971) that states that we are able to estimate a local inverse of a function if the determinant of the Jacobian is not zero. The estimation of the change in the policy parameter space is caused by a change in the task space is given by:

(10) $$\Delta \theta^{*}\approx J_{PS}\left(\tau^{*}\right)\Delta \tau^{*}$$

Since the parameterized skill is not a bijective mapping, multiple solutions can exist. We assume to sample in the local neighbourhood of our current estimate, therefore we initialize the gradient descent with PS(***τ***). Gradient descent is implemented by the Levenberg-Marquardt method (Liu and Han, 2003), also referred to as Damped Least-Squares method as depicted e.g., in (Buss, 2004), due to numerical stability in comparison to pseudoinverse and Jacobian transposed based methods. The incremental update of the estimated task space τ* is based on the Jaocbian ***J**_PS_*(τ*) of *PS* with respect to the input τ*:

(11) $$\begin{array}{rl} \Delta \tau^{*} & =J_{PS}(\tau^{*}{)}^{T}{\left(J_{PS}(\tau^{*})J_{PS}(\tau^{*}{)}^{T}+\lambda^{2}I\right)}^{-1}e\\ & with\;e=\left(\theta^{*}-PS(\tau^{*})\right) \end{array}$$

## 5. Evaluation on Toy Scenarios

**Figure 8:**
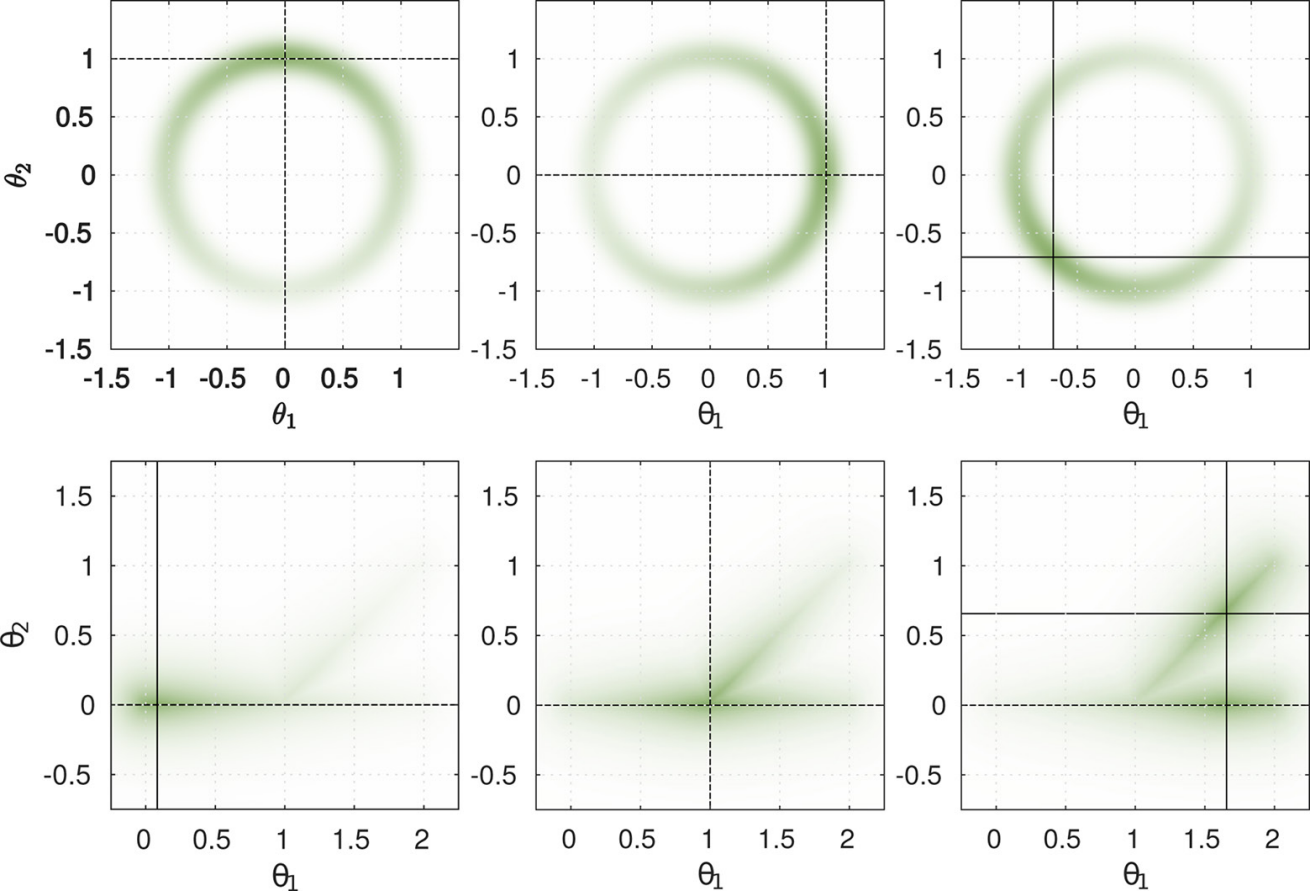
Visualization of designed reward functions. Circular reward unction *R*_circular_ (top) and branch reward *R*_branch_ (bottom) for three different task parameterizations are shown. Crossing points of horizontal and vertical black lines indicate maxima of reward functions. For *τ* > 1 multiple maxima of the reward function exist (bottom-right). Colour intensity indicates magnitude of reward for depicted parameterization ***θ***

To gain insight into the proposed hybrid search method, we investigate two test cases. For simplicity and visualization purposes, the policy is defined as the Identity and the reward function operates directly on $\theta \in R^{2}$ , the 2D space of the policy parameterization. The reward function is parameterized by $\tau \in R^{1}$  defining the position of maximum reward in the 2D space. This allows us to plot the reward function in relation to a fixed value of *τ*. A visualization of both reward functions for several fixed parameterizations *τ* are shown in Figure 8. The color intensity encodes the reward for a given task parameterization ***θ***. The first scenario describes a circular manifold with a maximum at **m***_τ_*, reward is given by:

(12) $$\begin{array}{c} R_{\text{a}}\left(\theta ,\;\tau \right)=\frac{1}{\sqrt{2\pi \sigma_{a}^{2}}}exp-\frac{|atan2\left({\text{m}}_{\tau }\times \theta ,\right){\text{m}}_{\tau }\cdot \theta |}{2\sigma_{a}^{2}}\\ R_{r}\left(\theta \right)=\frac{1}{\sqrt{2\pi \sigma_{\text{r}}^{2}}}exp-\frac{{\left(1-\|\theta \|\right)}^{2}}{2\sigma_{\text{r}}^{2}},{with\;\text{m}}_{\tau }=\left[\begin{array}{c} sin\left(\tau \right)\\ cos\left(\tau \right) \end{array}\right] \end{array}$$

The reward function includes the angular deviation *R*_a_(***θ***) as well as the deviation in the radius *R*_r_(***θ***), which are weighted by Gaussian functions. The overall reward is given by *R*_circular_(***θ***, *τ*) =*R*_a_(***θ***, *τ*) · *R*_r_(***θ***).

The second reward function is based on a branch manifold. For parameterizations *τ* ≤ 1 the maximum reward is located at [*τ*; 0]. For *τ* > 1 two maxima can be found at [*τ*; 0] and [*τ*; 1 + *τ*]. It is based on a combination of the distances to the parametrized maxima of the function *R*_m_(***θ***, *τ*) and the distance to the branch manifold *R*_b_:

(13) $$\begin{array}{rl} & R_{\text{m}}(\theta ,\tau )=\frac{1}{\sqrt{2\pi \sigma_{\text{d}}^{2}}}\exp-\frac{d_{\text{min}}}{2\sigma_{\text{d}}^{2}},\text{ with }\\ & d_{\text{min}}=\left\{ \begin{array}{ll} ||\theta -\left[\tau ;0\right]||, & \text{if }\tau \leq 1\\ min(||\theta -\left[\tau ;0\right]||,||\theta -\left[\tau ;\tau -1\right]||), & \text{else} \end{array}\right.\\ & \text{and}\;R_{\text{b}}(\theta )=\frac{1}{\sqrt{2\pi \sigma_{\text{r}}^{2}}}\exp-\frac{Dist_{\text{branch}}(\theta )}{2\sigma_{\text{r}}^{2}} \end{array}$$

**Figure 9:**
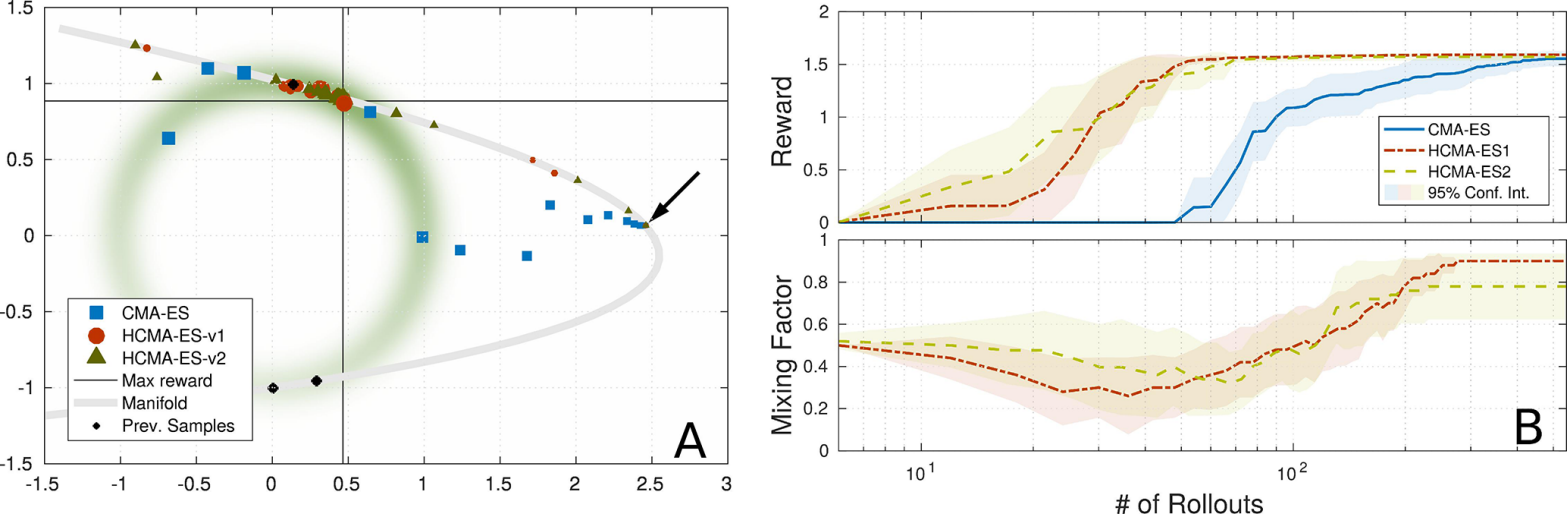
Comparison of optimization algorithms on 2D reward function: Overshoot of PS, hybrid optimization is able to utilize manifold of the parameterized skill (gray line) to perform optimization in 1D space. **(A)** Estimated means of algorithms during optimization, marker size indicates generation. Black arrow points to initial guess on manifold (gray line) of parameterized skill. **(B)** Comparison of reward and mixing factor during optimization is shown.

**Figure 10:**
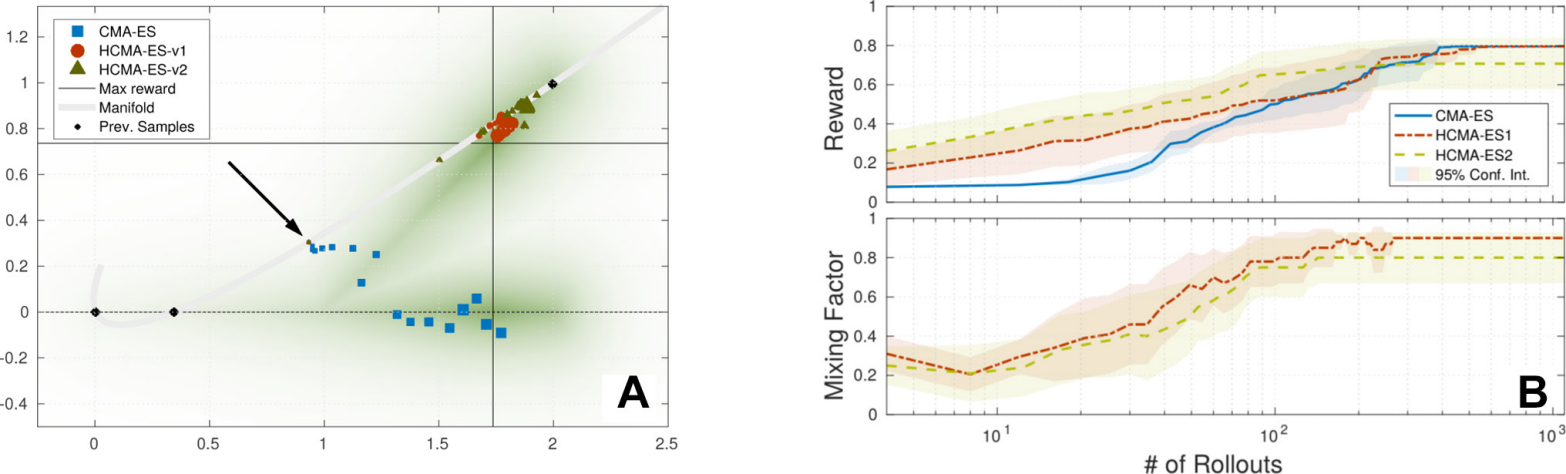
Comparison of optimization algorithms on 2D reward function: Multiple maxima of reward function. **(A)** Estimated means of algorithms during optimization, marker size indicates generation. Black arrow points to initial guess on manifold (gray line) of parameterized skill. **(B)** Comparison of reward and mixing factor during optimization is shown.

With *Dist*_branch_(*θ*), the minimum distance of *θ* to the line segments [0; 0] – [2; 0] and [1; 0 – 2; 1]. The combination of both reward terms results in the final reward function *R*_branch_(***θ***, *τ*) = *R*_m_(***θ***, *τ*) · *R*_b_(***θ***). We designed the scenario to reflect expected real world problems: The space of all possible actions includes a subset of appropriate actions on a manifold that have have higher rewards. Within this subset we expect a maximum of the reward function at parameterizations that solve the task in an appropriate way. We evaluated three cases as shown in Figure 9 to Figure 10. Each plot shows the comparison between a search in the policy space by CMA-ES as well as the behavior of our proposed hybrid algorithms. The green color intensity encodes the reward for a depicted policy parameterization ***θ***. Previous training data (obtained by estimation of one maximum of the reward function) of the parameterized skill is indicated by a black dot (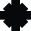). Based on the training data, the mapping *f*_PS_ on the manifold in the policy space, i.e., PS(*τ*), is constructed and shown as a gray line. The symbols 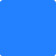, 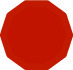 and 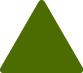 represent the current estimates of the means ${\text{m}}_{F}^{g}$ in the policy space, whereas the size 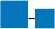, 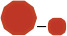 and 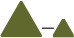 show the history of previous mean estimates ${\text{m}}_{F}^{g-n},\;\forall n\in \left\{1,\ldots ,\;g\right\}$ up to the first generation with decreasing size of the symbol. The real maximum of the reward function is marked by black crossing lines and the location of the initial estimate ***θ**_start_* = PS(*τ_i_*) on *f*_PS_ is highlighted by a black arrow (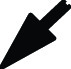).

In the following we will describe all three scenarios and the optimization process in detail:

### 5.1. Overshoot

The scenario in Figure 9 shows a situation in which an overshoot of the estimation of the parameterized skill occurs. We utilize the circular reward *R*_circular_ and perform an exponential distortion *f*(*τ*) =*exp*(*τ*) * *π* / exp(*π*) of the parameterization to enforce a generalization error of the memory resulting in *R*(***θ***, *τ*) =*R*_circular_(***θ***, *f*(*τ*)). For training of the parametrized skill we estimate optimal parameterizations ***θ**_i_* for three different tasks *τ_i_*.

For the depicted task parameterization, the parameterized skill proposes a solution that is located in a region with a little gradient information. By following the low dimensional embedding of the parameterized skill the hybrid approach is able to guide the optimizer into a region with stronger gradient and that is closer to a desired maximum of the reward function. In case of the original CMA-ES approach, it takes longer to reach a region with more informative gradient information and requires therefore more rollouts in comparison to the hybrid optimization in both spaces. But the evaluation of this scenario not always leads to a faster convergence of HCMA-ES-v1 and HCMA-ES-v2 in comparison to CMA-ES. In cases, where the estimate of the parameterized skill is of very low quality, optimization in the low dimensional space of *f*_PS_ can lead to a fast convergence to a region with higher rewards (as in Figure 9). But the algorithm could end up in an area that is far away from the final solution, so that an optimization by CMA-ES can reach a high reward with less number of executed rollouts. This results in a fast rising reward at the beginning of the hybrid optimization process followed by a period with a slowly rising reward as the estimate moves along the manifold towards the optimum of the reward function.

Algorithm HCMA-ES-v1 and HCMA-ES-v2 show comparable performance and a similar behavior during optimization. Investigations of the shape of the covariance reveal the extended update policy of HCMA-ES-v2 . Since the shape and size of the covariance of the policy space integrates rollouts sampled in the task space as well, the covariance grows and shapes aggressively into the direction of the real maximum and the shape of the manifold of the parameterized skill. Close to the maximum, i.e., the covariance shrinks but keeps the shape influenced by the previous fast approaching phase in the low dimensional manifold. Figure 9B shows the probability of performing a rollout in the policy parameter space, starting at equal probabilities for both spaces, the algorithm first shifts its focus to the task space and switches to a fine-tuning at the end of the optimization phase.

### 5.2. Multiple Minima

This scenario explores tasks with multiple solutions for a certain range of tasks parameterizations. This time we utilize the circular reward *R*_branch_ in combination with the exponential distortion *f*(*τ*) =*exp*(*τ*) * *π* / exp(*π*) used in the overshoot scenario. Therefore the reward function is given by *R*(***θ***, *τ*) =*R*_branch_[***θ***, *f*(*τ*)]. The presented training samples for the parameterized skill as well as the experimental setup can be seen in Figure 10. As discussed in Sec. 2, multiple maxima of the reward function bear the risk of generating inconsistent training data for the parameterized skill and impede generalization capabilities. It is beneficial to prefer solutions for tasks that are close to the manifold of the parameterized skill, in this case solutions on the upper branch of the reward function in Figure 10A. A hybrid optimization that performs a search along the manifold of the parameterized skill enhances the probability to find an optimum close to the manifold of the already established parameterized skill. As shown in Figure 10A,B, starting from the initial guess, the standard CMA-ES approach follows the gradient towards the manifold of the reward function. The covariance, responsible for perturbation of sampling, starts to shape into that direction and causes the optimizer to follow the gradient toward the lower branch of the reward function. The estimated solution is far off the manifold of the parameterized skill and would result in inconsistent training data since previous training data was selected from the upper branch. The standard CMA-ES optimization is able to find a solution for the given task without requiring a significant different number of rollouts than the hybrid optimization methods.

Although the hybrid search can not speed up the optimization process, the optimizer first prefers the manifold of the parameterized skill to move towards the gradient of the reward function, as shown in Figure 10B. For the final phase, the optimizer prefers the policy parameter space for optimization and is able to find a maximum of the reward function that is consistent with previous training data since it is located in the upper branch of the manifold of the reward function.

### 5.3. Results

As shown in Figure 9 to Figure 10, we were able to identify two situations in which our proposed hybrid CMA-ES algorithm is able to speed up optimization significantly. The mean rewards obtained for a given number of rollouts indicate a slightly faster convergence of HCMA-ES-v2 , but we can not observe a strong significant difference between HCMA-ES-v1 and HCMA-ES-v2 for those simple optimization tasks. We show that in case of a faulty estimate of the parameterized skill in a region with low gradient information the hybrid optimization scheme allows a faster convergence. The later experiment identifies a situation in which the consistency of the parameterized skill can be enhanced by a preference of solutions close to the previously established manifold.

## 6. Evaluation on Robotic Scenarios

**Figure 11:**
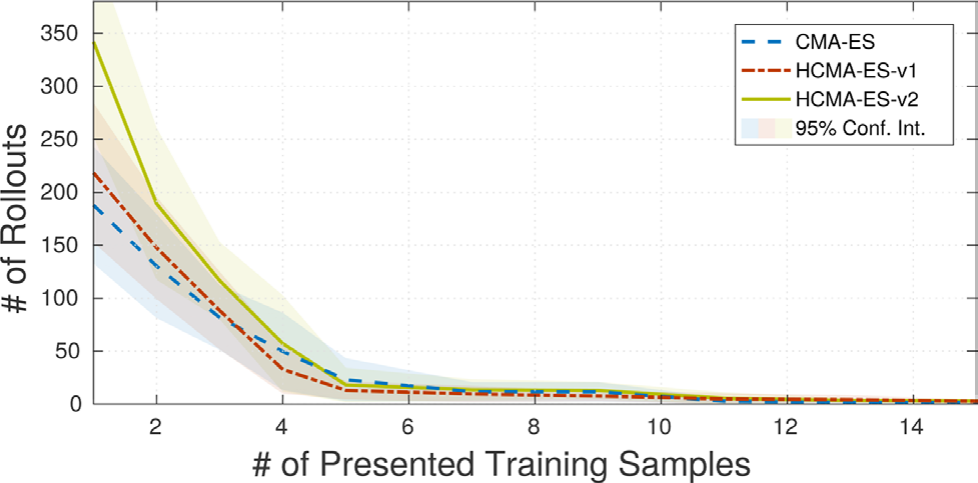
Results of the comparison of HCMA-ES to optimization in the policy parameter space for the point reaching scenario. It can be seen, that the number of required rollouts for task fulfilment is not significantly reduced by one of the optimization techniques.

The evaluation of the hybrid optimization scheme as proposed in Sec. 4 refers to the previously performed experiments as described in Sec. 3. We compare the original CMA-ES search in policy space to our hybrid search algorithms. To be able to compare the algorithms without the effect of different states of the memory we stored the memory states during performing the experiments in section Sec. 3.4. In the following experiments, we replicate the same conditions and replace the optimization algorithm by our proposed hybrid spaces optimization methods. Figure 11 shows the results of the 10-DOF planar arm scenario. HCMA-ES-v2 requires slightly more rollouts for task completion than HCMA-ES-v2 and plain CMA-ES in case the memory has been trained with less than 4 samples. This is caused by updating the covariance matrix of the policy space based on rollouts in task space. To reduce the overhead of the hybrid search algorithms, the initialization of *p*_E_ and *p*_F_ plays a crucial role. It can be expected that a search in the policy space is more beneficial as long as the number of training samples for the parameterized skill is low. No substantial difference between the CMA-ES and the hybrid search can be seen in the case the parameterized skills consolidated more than 4 samples, The update policies of HCMA-ES-v1 and HCMA-ES-v2 do not lead to significantly different results.

**Figure 12:**
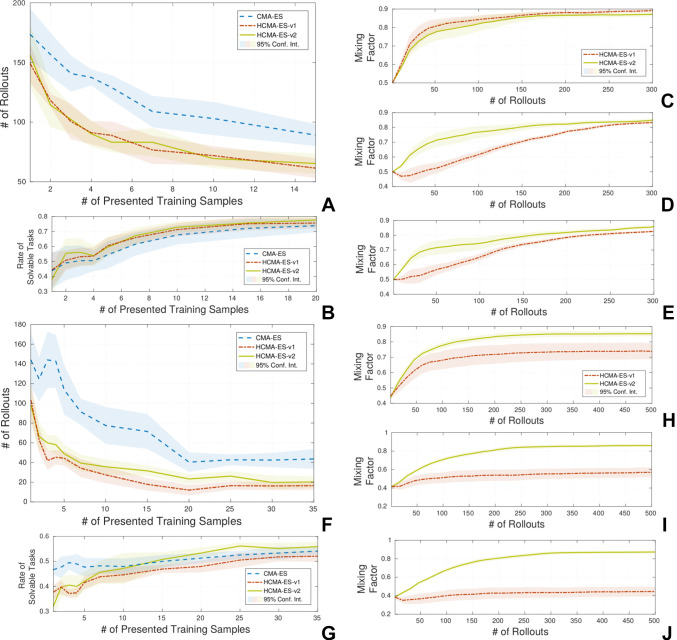
Results of the comparison of HCMA-ES to optimization in the policy parameter space for the point reaching scenario. Experiments **(A-E)** show results in end-effector space and **(F-J)** in joint space It can be seen, that the number of required rollouts for task fulfilment is significantly reduced by the proposed hybrid optimization technique** (A+F)**. The success rate of the optimization process (i.e., exceed a certain threshold on reward) shows stays the same compared to the optimization on the policy parameter space **(B+G)**. In **(C-E; H-J)** the behaviour of the mixing factor between the search spaces is shown for 1**(C+H)**, 5**(D+I)** and 20**(E+J)** training samples.

**Figure 13:**
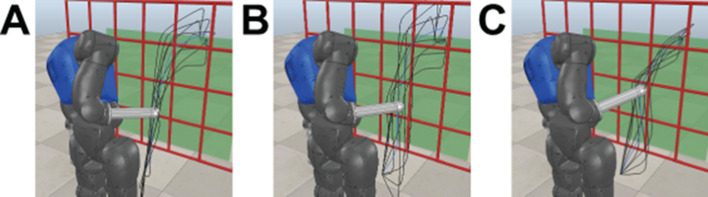
COMAN robot during execution of a estimated end-effector trajectory (blue) of the parameterized skill PS(***τ**_i_*) for one fixed reaching target ***τ**_i_*. Black trajectories visualize the variability in low dimensional search space ±50% of the input range PS(***τ**_i_ + **δ***_±50%_). From left to right, different states of the memory are shown (3,5 and 10 training samples).

The results for the second scenario, Sec. 3.4.2, show that the proposed hybrid search is able to reduce the number of required rollouts for solving unseen tasks as expected. The parameterized skill of the joint space experiments requires more training samples due to the lack of the inverse Jacobian controller that copes with distance maximization. The results are shown in Figure 12, (A-E) show results for experiments in end-effector space in the same way as (F-J) show results for joint space. Both hybrid optimization methods show a tendency to exceed the rate of solvable tasks of the standard CMA-ES method for the experiments in the end-effector space Figure 12. The results of the joint space experiments the are not that clear Figure 12. The different update policies of HCMA-ES-v1 and HCMA-ES-v2 can be seen by a comparison of the development of the mixing factors *p*_F_ in Figure 12(C-E;H-J) for 1, 5 and 20 presented samples to the parameterized skill. In case the parameterized skill has a good representation, HCMA-ES-v1 switches to an optimization in the policy space at a later stage Figure 12(E+J), whereas HCMA-ES-v2 clearly prefers the policy space for optimization. Both algorithms are switching to a search in the policy space in case of a low number of training samples and in case the memory has seen a certain amount of training samples, HCMA-ES-v2 supports a faster switching from task to policy space search. The visualization of the variance in the low dimensional parameter space is shown in Figure 13. We compare three different states of the parameterized skill by plotting estimated solutions for variations of the input around the current task parameter. We can observe different strategies of the robot like approaching the target point from top or from bottom.

## 7. Discussion and Conclusion

We were able to identify two situations in which we expect our algorithm to exceed the performance of an optimization in policy space, as discussed in Sec. 5. We created three test scenarios, in which we were able to show the benefits of the proposed algorithm as well as one case in which our algorithm underlays a plain policy space search. We could see a clear advantage of the proposed hybrid optimization although the reduction ratio of the task space to the policy parameterization is only 2:1 for these idealized test cases. The scalability of our proposed method was evaluated in complex robot scenarios. We were not able to show significant performance improvements of the hybrid search for the optimization of a 10-DOF robot scenario, while an optimization of a point reaching task of a humanoid robot showed the expected advantages of our approach. We believe that the design of the 10-DOF reaching task, e.g., no obstacles, results in a simple reward function in the high dimensional policy space. The optimizer in the full policy space is able to follow the gradient efficiently after initial estimation of the covariance, e.g., direction, and a reduction of the search space is not necessary. In such a situation, our algorithm is not able to exploit the benefits of the low dimensional embedding of the parameterized skill and has to cope with overhead produced by the combination of both spaces. The skill learning is faced with a much more complex optimization problem, in case of the humanoid robot reaching task, like joint limits and obstacle constraints. Even not all requested task instances are solvable by the kinematics of the robot and CMA-ES can not solve all tasks as it gets stuck in local minima. We are able to show the benefits of our proposed combined optimization scheme for this complex scenario. Although the evaluation was limited to reaching tasks, we demonstrated the applicability of the approach in different domains by an evaluation of control in joint and Cartesian space. An extension to rhythmic movements can be achieved by modification of the underlying DMP representation (Ijspeert et al., 2002). Due to the modular design of the framework other policy representations, black-box-optimizer and learning algorithms can be integrated. One crucial benefit of the point-attractor representation of the DMP is the linearity of its parameterization in relation to the task parameterization (e.g., target position). In comparison to e.g., vector field representations, instabilities can be avoided and the dimensionality of the policy parameterization is reduced. The system is designed to rely on the results of the optimization process, therefore it has no implicit capabilities of dealing with multiple objectives, like in e.g. (Pirotta et al., 2015; Parisi et al., 2017). The pre-designed reward function has to reflect appropriate goals to fulfil the range of parameterized task instances. Policy estimation for multiple objectives can only be achieved by an encoding of the relevance of the objectives as task parameterization.

### 7.1. Conclusion

We propose the exploration of a parameterized skill by an extension of the CMA-ES optimization to hybrid spaces. We evaluate scenarios in which we are able to observe a low dimensional parameterization for a new task instance. By consolidation of found solutions and their parameterizations of previous tasks we are able to incrementally learn a parameterized skill. The parameterized skill is able to generalize for new policy parameters from task parameterizations, resulting in better start configurations of the optimizer or in the optimal case in a sufficient solution without further optimization. A hybrid search is performed in the space of the policy parameters as well as in the low dimensional manifold that is generated by the parameterized skill in case further optimization is necessary. We have been able to identify and verify several scenarios in which this hybrid approach shows a faster convergence. Additionally we evaluate our approach on complex robotic scenarios targeting on end-effector and joint space control. Our results show that for high task complexity, i.e., upper body reaching, our proposed hybrid optimization is able to significantly speed up policy optimization.

## Author Contributions

JQ: Conception and design of the research, acquisition, analysis and interpretation of data; Writing of the paper. JS: Conception and design of the research, analysis and interpretation of data; Writing of the paper.

## Conflict of Interest Statement

The authors declare that the research was conducted in the absence of any commercial or financial relationships that could be construed as a potential conflict of interest.

## Appendix A

### Appendix A: Details of CMA-ES Update

Sampling from a multivariate normal distribution centered at the current estimate:

(A.1) $$\begin{array}{rl} {\text{x}}_{k}^{\left(g+1\right)}= & {\text{m}}^{\left(g\right)}+\sigma^{\left(g\right)}{\text{y}}_{k}^{\left(g+1\right)}\;\text{with}\\ & {\text{y}}_{k}^{g+1}∼N_{k}(0,({\text{C}}^{\left(g\right)})\;\text{for }k=1,...,\lambda \end{array}$$

Update of mean m(g+1) given by:

(A.2) $${\text{m}}^{\left(g+1\right)}={\text{m}}^{\left(g\right)}+\sigma^{\left(g\right)}{\text{y}}_{w}^{\left(g+1\right)},\;\text{with }{\text{y}}_{w}^{\left(g+1\right)}=\sum_{i=1}^{\mu }{\text{w}}_{i}{\text{y}}_{i:\lambda }^{\left(g+1\right)}$$

Where ${\text{x}}_{i:\lambda }^{\left(g+1\right)}$denotes the i-th best individual and the index i : λ denotes the index of the i-th ranked individual $R({\text{x}}_{1:\lambda }^{g+1})\leq R({\text{x}}_{2:\lambda }^{g+1})\leq \cdots \leq R({\text{x}}_{\lambda :\lambda }^{g+1})$. With covariance **C**(g)∈ ℝ^FxF^ scaled by σ(g)∈ ℝ_+_ for generation g. Update of the covariance matrix and its evolution path:

(A.3) $$p_{c}^{\left(g+1\right)}=(1-c_{c})p_{c}^{\left(g\right)}+\sqrt{1-(1-c_{c}{)}^{2}}\sqrt{\mu_{eff}}y_{w}$$

(A.4) $$\begin{array}{rl} C^{\left(g+1\right)}=(1-c_{1}-c_{\mu })c^{\left(g\right)} & +c_{1}p_{c}^{\left(g+1\right)}{p_{c}^{\left(g+1\right)}}^{⊤}\\ & +c_{\mu }\sum_{i=1}^{\mu }w_{i}y_{i:\lambda }^{\left(g+1\right)}{y_{i:\lambda }^{\left(g+1\right)}}^{⊤} \end{array}$$

Update of the scaling sigma and its assigned evolution path:

(A.5) $$p_{\sigma }^{\left(g+1\right)}=(1-c_{\sigma })p_{\sigma }^{\left(g\right)}+\sqrt{1-(1-c_{\sigma }{)}^{2}}\sqrt{\mu_{eff}}{C^{\left(g\right)}}^{-1/2}y_{w}^{\left(g+1\right)}$$

The operation performed by ^$C^{(g{)}^{\text{1/2}}}$^ results in a rescaling of the expected distance of samples to the center, as described in (Hansen (2006)), Eq. 23. The update of sigma is performed by: (A.6)

(A.6) $$\sigma^{\left(g+1\right)}=\sigma^{\left(g\right)}\times \exp\left(\frac{c_{\sigma }}{d_{\sigma }}\left(\frac{||p_{\sigma }^{\left(g+1\right)}||}{E||N\left(0,I\right)||}-1\right)\right)$$

## Appendix B

### Appendix B: Details of Hybrid CMA-ES Update

Update of the optimization by CMA-ES in hybrid spaces as refered to in Sec. 4.1. Update of the estimated means:

(B.1) $$\begin{array}{rlr} x_{k,E}^{\left(g+1\right)} & =m_{E}^{\left(g\right)}+\sigma_{E}^{\left(g\right)}y_{k,E}^{\left(g+1\right)}\; & \text{for }k=1,...,\lambda_{H}\;|\;s_{k}^{\left(g+1\right)}=0\\ x_{k,F}^{\left(g+1\right)} & =m_{F}^{\left(g\right)}+\sigma_{F}^{\left(g\right)}y_{k,F}^{\left(g+1\right)}\; & \text{for }k=1,...,\lambda_{H}\;|\;s_{k}^{\left(g+1\right)}=1 \end{array}$$

Interpolated number of rollouts per generation given by:

(B.2) $$\lambda_{H}=p_{E}\lambda_{E}+p_{F}\lambda_{F}=4+p_{E}\lfloor 3\ln\left(E\right)\rfloor +p_{F}\lfloor 3ln(F)\rfloor$$

Projection of samples by parameterized skill:

(B.3) $$\begin{array}{rlr} x_{k,E}^{\left(g+1\right)} & ={PS^{\left(g\right)}}^{-1}(x_{k,F}^{\left(g+1\right)})\; & \text{for }k=1,..,.\lambda_{H}\;|\;s_{k}^{\left(g+1\right)}=1\\ x_{k,F}^{\left(g+1\right)} & ={PS^{\left(g\right)}}^{-1}(x_{k,F}^{\left(g+1\right)})\; & \text{for }k=1,..,.\lambda_{H}\;|\;s_{k}^{\left(g+1\right)}=0 \end{array}$$

Update of probabilities to sample from embedded or full space:

(B.4) $$\delta p_{E}^{\left(g+1\right)}=\frac{\sum_{\begin{array}{c} k=1,\\ s_{k}^{\left(g+1\right)}=0 \end{array}}^{\mu }W_{k}^{\left(g+1\right)}}{\sum_{k=1}^{\mu }W_{k}^{\left(g+1\right)}}-\delta p_{E}^{\left(g\right)},\;\delta p_{F}^{\left(g+1\right)}=-\delta p_{E}^{\left(g+1\right)}$$

Rescaling of samples for update of covariance:

$$\begin{array}{rl} {\tilde{y}}_{w,E} & =\sum_{\begin{array}{c} i=1\\ s_{i:\lambda }^{\left(g+1\right)}=0 \end{array}}^{\mu }w_{i}y_{i:\lambda ,E}^{\left(g+1\right)}+\frac{\chi_{E}}{\beta_{E}}\sum_{\begin{array}{c} i=1\\ s_{i:\lambda }^{\left(g+1\right)}=1 \end{array}}^{\mu }w_{i}y_{i:\lambda ,E}^{\left(g+1\right)}\;\text{with} \end{array}$$

(B.5) $$\begin{array}{rl} \beta_{E}= & \sqrt{\mu_{eff}}\left\|C_{E}^{-1/2}\sum_{\begin{array}{c} i=1\\ s_{i:\lambda }^{\left(g+1\right)}=1 \end{array}}^{\mu }\frac{w_{i}y_{i:\lambda ,E}^{\left(g+1\right)}}{\alpha_{F}}\right\|,\;\alpha_{F}=\sum_{\begin{array}{c} j=1\\ s_{j:\lambda }^{\left(g+1\right)}=1 \end{array}}^{\mu }w_{j} \end{array}$$

With $\chi_{N}\overset{def}{=}\sqrt{E(\chi_{N}^{2})}=E||N(0,I_{N})||$ referring to the chi-squared distribution $\chi_{N}^{2}$ with N degrees of freedom.

(B.6) $$\begin{array}{rl} {\tilde{y}}_{w,F} & =\sum_{\begin{array}{c} i=1\\ s_{i:\lambda }^{\left(g+1\right)}=1 \end{array}}^{\mu }w_{i}y_{i:\lambda ,F}^{\left(g+1\right)}+\frac{\chi_{N}}{\beta_{F}}\sum_{\begin{array}{c} i=1\\ s_{i:\lambda }^{\left(g+1\right)}=0 \end{array}}^{\mu }w_{i}y_{i:\lambda ,F}^{\left(g+1\right)}\;\text{with}\\ \beta_{F}= & \sqrt{\mu_{eff}}\left\|C_{F}^{-1/2}\sum_{\begin{array}{c} i=1\\ s_{i:\lambda }^{\left(g+1\right)}=0 \end{array}}^{\mu }\frac{w_{i}y_{i:\lambda ,F}^{\left(g+1\right)}}{\alpha_{E}}\right\|,\;\alpha_{E}=\sum_{\begin{array}{c} j=1\\ s_{j:\lambda }^{\left(g+1\right)}=0 \end{array}}^{\mu }w_{j} \end{array}$$

With $\beta_{E}^{-1}$ responsible for a rescaling of samples from policy parameter space to task space and $\beta_{F}^{-1}$ from task parameter space to policy space.

And the update of the exploration width:

(B.7) $$\begin{array}{r} \sigma_{E}^{\left(g+1\right)}=p_{E}{\tilde{\sigma }}_{E}^{\left(g+1\right)}+p_{F}\frac{{\tilde{\sigma }}_{F}^{\left(g+1\right)}\beta_{E}}{\chi_{E}}\\ \sigma_{F}^{\left(g+1\right)}=p_{F}{\tilde{\sigma }}_{F}^{\left(g+1\right)}+p_{E}\frac{{\tilde{\sigma }}_{E}^{\left(g+1\right)}\beta_{F}}{\chi_{F}} \end{array}$$
